# Multipolar Effects in the Optical Active Second Harmonic Generation from Sawtooth Chiral Metamaterials

**DOI:** 10.1038/srep22061

**Published:** 2016-02-25

**Authors:** Huimin Su, Yuxiang Guo, Wensheng Gao, Jie Ma, Yongchun Zhong, Wing Yim Tam, C. T. Chan, Kam Sing Wong

**Affiliations:** 1 Department of Physics, The Hong Kong University of Science and Technology, Hong Kong, P. R. China; 2 Key Laboratory of Optoelectronic Information and Sensing Technologies of Guangdong Higher Education Institutes, Department of Optoelectronic Engineering, Jinan University, Guangzhou 510632, P. R. China; 3 Department of Physics, The South University of Science and Technology of China, Shenzhen 518055, P. R. China

## Abstract

Based on the facts that chiral molecules response differently to left- and right-handed circular polarized light, chiroptical effects are widely employed for determining structure chirality, detecting enantiomeric excess, or controlling chemical reactions of molecules. Compared to those in natural materials, chiroptical behaviors can be significantly amplified in chiral plasmonic metamaterials due to the concentrated local fields in the structure. The on-going research towards giant chiroptical effects in metamaterial generally focus on optimizing the field-enhancement effects. However, the observed chiroptical effects in metamaterials rely on more complicated factors and various possibilities towards giant chiroptical effects remains unexplored. Here we study the optical-active second harmonic generation (SHG) behaviors in a pair of planar sawtooth gratings with mirror-imaged patterns. Significant multipolar effects were observed in the polarization-dependent SHG curves. We show that the chirality of the nanostructure not only give rise to nonzero chiral susceptibility tensor components within the electric-dipole approximation, but also lead to different levels of multipolar interactions for the two orthogonal circular polarizations that further enhance the nonlinear optical activity of the material. Our results thus indicate novel ways to optimize nonlinear plasmonic structures and achieve giant chiroptical response via multipolar interactions.

Chirality is a basic characteristic of living matters. It has been widely studied in the field of biochemistry, biology, catalysis and pharmacology. Chiral object refers to a form lack of any planes of mirror symmetry and occurs as either left- or right-handed enantiomers. Characterization techniques to probe chirality are usually based on the facts that chiral molecules response differently to left- and right-handed circularly-polarized (LCP and RCP) light. Given many organic molecules are chiral and their handedness plays an important role in chemical reactions, chiroptical effects, such as optical rotation (OR) and circular dichroism (CD), have been serving as a spectroscopic tool for determining structural chirality, enantiomeric excess, or controlling the biological functions of complex molecules.

Though optical-activity effects tend to be rather weak in all natural materials, it has been demonstrated that giant chiroptical effects can be achieved in chiral plasmonic metamaterials^1^. With the excitation of surface plasmonic resonance (SPR), chiroptical effects can be significantly amplified by engineering the local field intensities and controlling the coupling between the electric and magnetic fields. Moreover, it has been demonstrated that plasmonic nanostructures can generate “superchiral” electromagnetic near field that further enhance the chiroptical interactions close to the nanostructure^2^,^3^,^4^,^5^. In addition to the linear chiroptical effects, nonlinear optical processes are also significantly enhanced in chiral metamaterials as compared to conventional nonlinearity in natural materials. In particular, optical second harmonic generation (SHG) often give rise to the largest nonlinear optical signal but is usually forbidden in centrosymmetry materials. As centrosymmetry is broken at surfaces and interfaces, the enormous electromagnetic field enhancement in metamaterials at the metal surfaces can boost the surface-sensitivity of SHG technique down to atomic monolayers^1^. Centrosymmetry is also broken by the presence of chirality, anisotropic SHG and SHG circular dichroism (SHG-CD) can thus serve as highly sensitive probes for exploring chiral structures. The second-order nonlinear chiroptical effects, i.e., optical rotation and circular dichroism in second harmonic generations (SHG-OR, SHG-CD), are typically much more pronounced than their linear optical counterparts^1^,^6^,^7^.

Giant chiroptical responses have been observed experimentally in chiral metamaterials built with various three-dimensional (3D)^8^,^9^,^10^,^11^ or quasi-planar nanoparticles^12^,^13^,^14^,^15^,^16^,^17^,^18^,^19^,^20^,^21^. Fabrications of full three-dimensional (3D) chiral metamaterials are challenging and time consuming, especially in the optical regime. In contrast, the so-called planar chiral metamaterials (PCM) are more compatible with planar process and easier to fabricate. It is also proposed that negative refraction can be realized in chiral nanostructures with strong chirality with neither the dielectric permittivity nor magnetic permittivity negative, which is more appealing from a practical point of view^22^. Compared to the linear chiroptical effects, the strong CD-SHG behaviors in metamaterials were usually attributed to the enormous electromagnetic field enhancement in the structure, confirmed by the fact that the SHG microscopy pattern matches very well with the distribution of hotspots at the fundamental frequency^23^. The ongoing research towards giant chiroptical effects generally focuses on optimizing the local field enhancement effects. However more complicated factors play a role in the SHG activity observed in the far-field, including the chiral symmetry-breaking of the structure, polarization properties of the local field, and the out-coupling efficiency at the second harmonics. More possibilities to obtain giant chiroptical response shall be investigated.

In this work, we employ planar sawtooth grating and its 2D mirror enantiomer to study the CD-SHG efficiency in chiral nanostructures. Large CD signals and asymmetric transmission have been observed in the visible range in our previous work^18^. We show that, for the case of these structures, the variation of SHG efficiency on the pump polarization contains direct evidence of significant contribution of bulk multipolar interactions. The chirality of the nanostructure not only give rise to nonzero chiral-symmetry-allowed nonlinear susceptibility tensor components, but also leads to different levels of effective multipolar interactions that contribute to the CD-SHG effects. Thus it allows for the possibility to further enhance the nonlinear chiroptical response of nanostructures by optimizing their chiral symmetry.

## Results

### Chiral nanostructures and their linear optical properties

**Figure 1:**
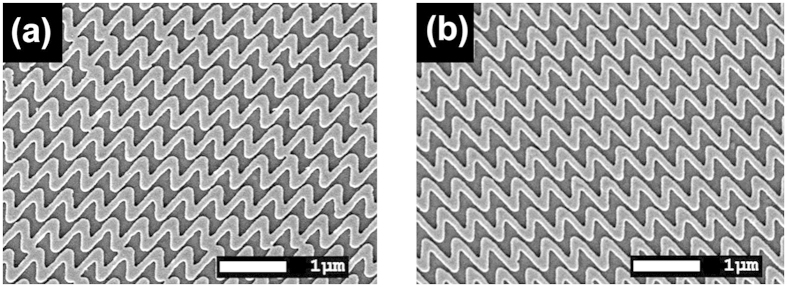
SEM images of (**a**) И-type (mirror-N) and (**b**) N-type sawtooth grating viewed from the top of air/grating/ITO substrate before embedding.

**Figure 2:**
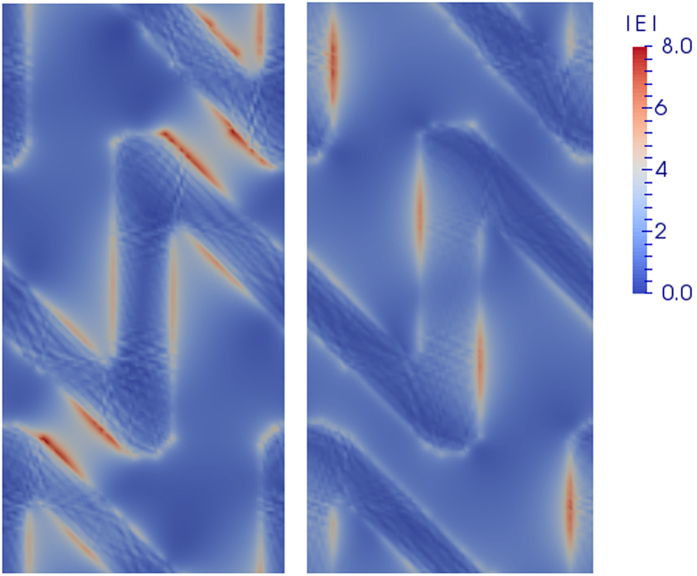
Calculated distribution of the local electric field magnitude |***E***| on the plane 5 nm after the N-type Au grating by backward excitation (k along -z direction) with LCP (left) and RCP (right) waves at the fundamental frequency (820 nm).

The Au sawtooth gratings of 50 nm in thickness are shown in Fig. 1. The И and N unit patterns are 450 × 450 nm^2^ in size and shifted with half lattice spacing horizontally (in the *x*-direction) between neighboring rows. The width of the sawtooth is ~110 nm. We define the N-type and mirror-N type sample by the patterns viewed from the air/grating side of the air/grating/substrate structures (Fig. S1). We also define the forward and backward excitations as the incidence of laser on the grating/substrate interface and the one on the air/grating interface, respectively. Previous work showed the mirror-N (И-) and N-patterned Au sawtooth gratings exhibit large CD signals in the range of 600–900 nm due to the difference in the Ohmic losses in the metal for the LCP and RCP incidence light^18^. The forward and backward CDs in each samples are antisymmetric, implying their “2D chirality”^18^. Furthermore, the transmittance, reflectance, and absorption measurements show the И- and N-patterned gratings are a pair of enantiomers in mirror forms^18^. Near-field calculations on the N-type grating with backward 820 nm excitation shows that higher field concentration can be obtained upon LCP illumination in the nanogaps between the neighbouring rows of the grating lines (Fig. 2). However negative CD signals were detected in this sample due to the higher ohmic power loss upon RCP illumination at this wavelength (Figs S2 and S3).

### Optical active second harmonic generation

**Figure 3:**
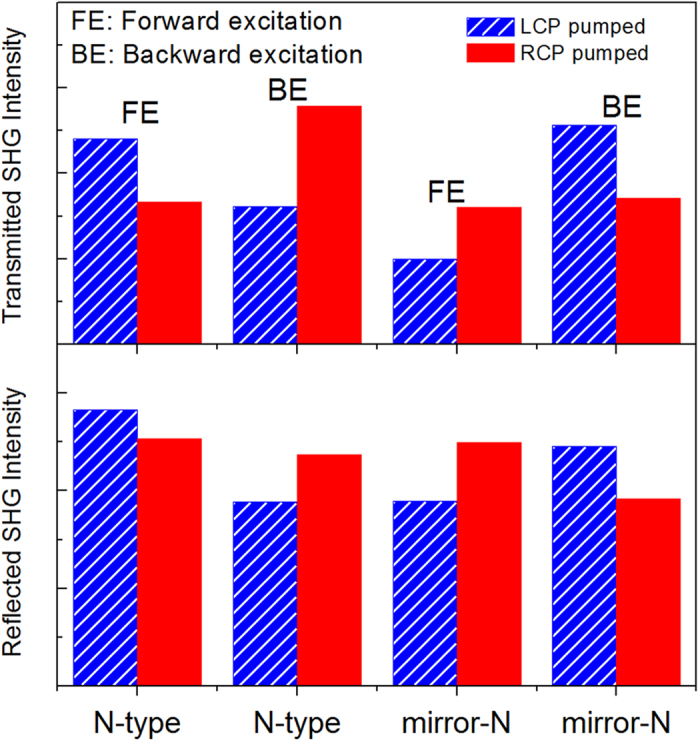
Measured SHG signals from the N and mirror-N gratings with circularly polarized fundamental laser at 820 nm (60 mW) in transmission (up) and reflection (down) geometry.

To characterize the SH responses of the sawtooth gratings, different combinations of excitation and input polarization were implemented. The intensity of SHG signals shows a quadratic dependency 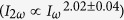 on the pump laser power (Fig. S4). The comparison results on the output intensity of LCP- and RCP-pumped SHG are summarized in Fig. 3. Both the N-type and mirror-N samples show optically-active SH responses, i.e., the non-zero 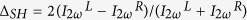, but with opposite signs in each cases. Upon forward excitation, for example, LCP fundamental beam generates stronger SHG signal in the N-type grating than the RCP wave, while in the mirror-N grating, a weaker SH response was detected with LCP illumination compared to the RCP illumination case. Moreover in both gratings, the observed SH optical activity have opposite behaviors upon forward and backward excitation, analogue to the asymmetric transmission phenomenon observed in the linear optical process^18^. Finally, the sign of CD-SHG-effects does not rely on the detection geometry of the SHG signals, suggesting the SH radiation is still dominated by the dipole-type response^24^.

Results in the Fig. 3 not only verify the 2D chirality of the gratings in the nonlinear optical domain, but also indicate that the local-field effect on the metal surface is not the only factor for effective SHG response in the structures. According to Fig. 2, one may expect that in the N-type gratings the generation of “hot spots” with higher local-field intensity upon LCP illumination would lead to enhanced SH emission compared to the RCP illumination case under backward excitation. However, our results suggest the presence of the hot-spots does not necessarily result in stronger SH activity in the far-field. On the one hand, out-of-phase SH signals generated at individual “hot-spots” may cancel each other in the far-field^25^,^26^,^27^. On the other hand, the local-field distribution in metamaterials is highly inhomogeneous, which could enhance coupling to multipolar effects^24^,^28^. The multipole (magnetic dipole and electric quadrupole) interactions allow second-order nonlinear response in the whole materials, which have different behaviors from the SH emission arising from surface dipoles^7^,^24^,^29^. They also become more important when the dimensions of the nanostructures are comparable to the wavelength of the incident light. Finally, the energy dissipation in the metal and the outcoupling efficiency of various kinds of SH sources also account for the overall far-field SH response.

**Figure 4:**
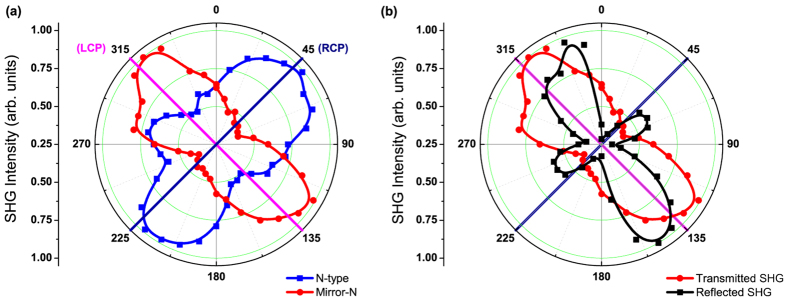
The SHG intensity as a function of the quarter wave plate (QWP) angle (backward excitation). (**a**) Transmitted SHG signal from the N (blue squares) and the mirror-N (red circles) grating. (**b**) Transmitted SHG (red circles) and reflected SHG (black squares) signal from the mirror-N grating. The polarizer before the QWP was oriented along the vertical direction (0 degree of the QWP angle). The angles highlighted in navy and magenta colors represent the RCP and LCP excitation respectively. The two-end arrow with dash line indicates the phase shift between the transmitted SHG and the reflected SHG.

To obtain direct evidence of multipole contribution to the far-field SH response, we measured the polarization-dependent SHG signals from the N and mirror-N gratings in both reflection and transmission geometry. The polarization state of the fundamental beam was controlled with a Glan polarizer and a quarter wave plate (QWP). Figure 4 compares the transmitted or reflected SHG intensities as functions of the QWP angles in either the N or the mirror-N gratings. All the curves have been normalized to the maximum intensity obtained in each configuration. The SHG signals measured in the gratings show clear polarization dependence on the excitation field against the in-plane chirality of the nanostructured metal surfaces. The highest SHG efficiency occurs as a consequence of surface charges oscillating resonantly with the pumping field, which is consistent with the linear CD measurement and ohmic loss calculation performed on the gratings (Figs S2 and 3). The transmitted SH radiation from the N-type grating reached its maximum when pumped with RCP fundamental light. On the contrary, the RCP-pumped SHG signal from the mirror-N grating was only 40% of those for the LCP case. Compared to the linear optical activities, the relatively larger contrasts between the two circular polarizations make the SHG measurement an ideal candidate for the detection and classification of chiral nanostructures. Within the electric dipole approximation, the intensity patterns of the SHG emission from the two structures should be related to each other by the reflection symmetry about the 0° plane. However the line shapes of the SHG signal display a significant asymmetry between the two structures (Fig. 4a), implying the achiral (bulk) multipolar contributes to the SH response in additional to the chirality-dependent surface dipole term^7^,^30^. The reflected SHG signals from both gratings were also measured at different pumping polarizations. The SHG efficiency lines exhibit different angular patterns and a significant phase shift between the two detection directions (Fig. 4b), indicating considerable multipole contribution to the SHG response of the structures. Different from the radiative property of electric dipoles, the electric field ***E*** of the magnetic dipole or electric quadrupole change in sign between the forward and backward radiation directions in the far field. Hence different radiation patterns will be observed in the transmitted and reflected directions, originated from the interference between various types of SH sources generated at different pumping polarizations^30^,^31^. As a consequence the divergence of SHG efficiency line shape between the two observing directions allows us to distinguish the multipolar contribution to the overall SHG response apart from those of the electric dipole.

## Discussion

To analyze the multipole effect, the nonlinear response tensor (NRT) approach^24^,^32^,^33^ was applied to fit the line shapes of the SH signal to the polarization components of the fundamental field in each configuration^34^:


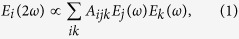


where *ijk* refer to the Cartesian coordinates in the laboratory frame. The coefficients *A*_*ijk*_ are linear combinations of the macroscopic nonlinear susceptibility tensor components taking into account electric-dipole only interactions 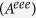, multipolar interactions at the fundamental frequency 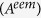, and multipolar interactions at the SH frequency 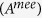
7. Both gratings have the chiral surface with 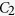 symmetry. The nonvanishing tensor components are *zzz*, *zxx*, *zyy*, *zxy* = *zyx*, *xxz* = *xzx*, 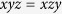, 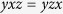, 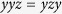, where *z* is the surface normal. The first 5 terms of electric or/and magnetic interactions do not contribute to the far-field SHG signal since the polarization of the resulting second-harmonics directed along the direction of the wave vector of light and cannot propagate in the forward or backward direction. Accordingly, the output radiation field at the second-harmonic can be expressed as:


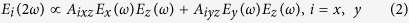


where the expansion coefficients 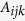 for various experimental geometries are shown in Table S1.

**Figure 5:**
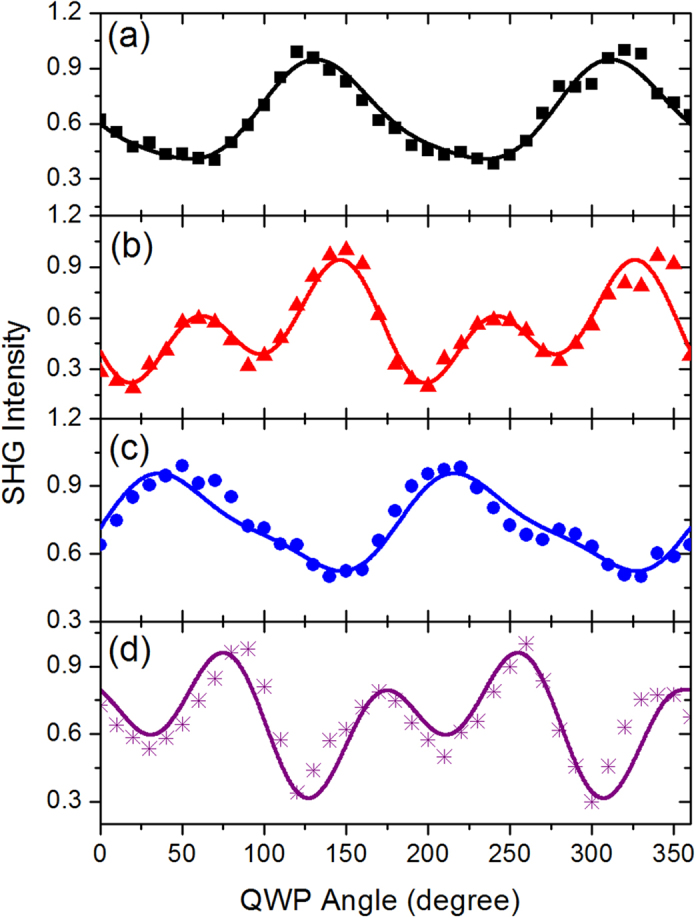
SH intensities generated in the transmission direction (**a,c**) and reflection direction (**b,d**) for the mirror-N (**a,b**) and N-type (**c,d**) structure as functions of the rotation angle of the quarter-wave plate. The points represent the experimental data, and the solid lines are the fits to the nonlinear response tensor (NRT) model.

The experimental results were then normalized to unity and fitted to the NRT model of equation (2). The best fits are shown in Fig. 5 and Table.1 lists the best fit of the isotropic combinations of tensor components for the mirror-N sample relative to 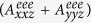. As shown in Table. 1, the SH response is dominated by the magnetic tensor components, and contribution of the pure electric dipolar components is around 35%. The metal grating itself in these samples possess mirror symmetry for the mirror perpendicular to the two-fold rotational axis 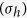. Hence the bulk SHG from pure electric dipole interactions vanish and the surface electric-dipole interactions on the upper and lower metal surface cancelled each other to a certain extent. It results in relatively low surface dipole contribution to the SHG signal and weak local-field enhancement effects on the metal surface. The multiple interactions, on the other hand, become more efficient due to the highly inhomogeneous field across the nanoscale structures.

**Figure 6:**
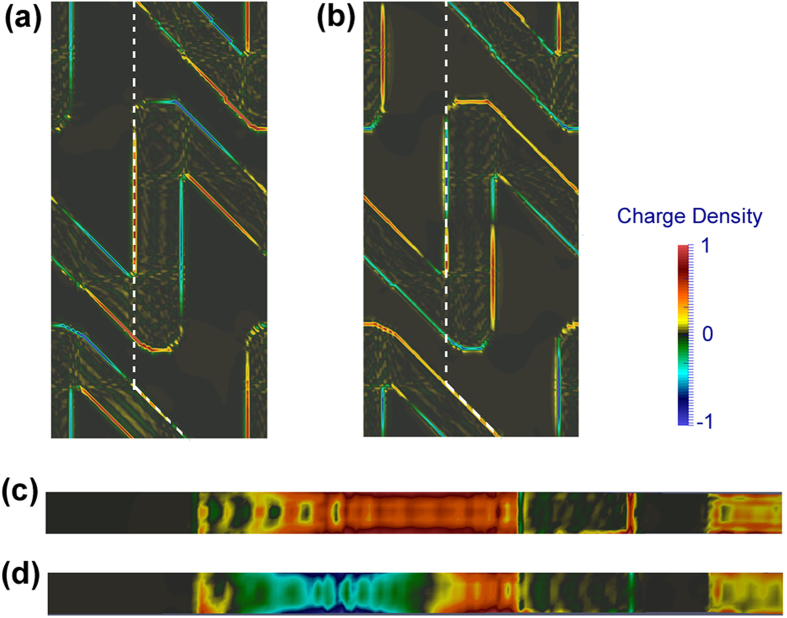
Calculated surface charge distribution of N-type grating upon backward (along –*z* direction) LCP (**a,c**) and RCP (**b,d**) illuminations. (**a,b**) are the charge distributions on the Au-grating/glass interface (XY-plane). (**c,d**) are the charge distributions on the side surfaces of the Au sawtooth as indicated by the white dashed lines in (**a,b**).

Among all these combinations, 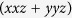, 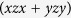 of the magnetic tensors and 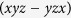 of the electric dipole tensor are associated with chirality and responsible for the CD effects in the second-order nonlinear optical process^7^. All the nonvanished tensor components contribute to the nonlinear response of the gratings, but only the out-of-phase parts between the chiral and achiral coefficients give rise to the chiral signature and circular differences in our experiment. The largest contribution to the CD-SHG signal comes from the multipolar interactions at the fundamental wavelength 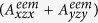. As shown in Fig. 6 for the N-type structure, the asymmetry surface charge distribution upon RCP fundamental wave could give rise to effective quadrupole sources 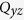 and 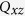 in the structure that lead to the far-field radiation along the *z*-direction. Upon LCP fundamental wave on the contrary, the microscopic nonlinear dipole sources locate more symmetrically on the upper and lower metal surfaces and thus reduce the radiation of quadrupole sources along the surface normal. As a result, the far-field SHG response was lower for the LCP case although it contains “hotter” spots in the nanogap between the neighboring rows of the sawtooth chain. We suggest the retardation of the electromagnetic field across the nanostructure gave rise to the multipolar terms in the nonlinear response of the gratings, while the relative oscillation strengths and phases between different multipolar sources depended on the polarization of the driven field. It modified the coupling efficiency of the incident field to different multipolar components and also led to different second-harmonic radiation intensity in the far field. Our experiments demonstrate that the CD-SHG response in chiral nanostructure can not only be enhanced by the field concentration effects but also by the difference in level of the higher multipolar interactions evoked by the two circular polarizations. It suggests another possible direction in optimizing the plasmonic structures for giant chiroptical effects. Compared to the surface-like electric dipole interactions, a large portion of multipolar response belongs to the bulk-type effects. Enhanced multipolar contribution may be implicated in thicker films or developing 3D helical structures or multi-layered materials with twisted unit structures. But the thickness of the nanostructures should be carefully chosen to balance the increasing of bulk effects and the decreasing of field retardation effects for thicker films^35^,^36^. Note that another type of multipole effects arising from atomic-level light-matter interactions can also occur in nanostructures and contribute to the nonlinear optical response of various samples^29^. However for the sawtooth gratings, neither *x*- or *y*-polarized EM field can drive an effective magnetic field that lead to a transverse SHG component. Hence the interactions with magnetic dipolar moments are less important in our experiments.

In summary, we have observed up to 60% difference in SHG from the planar sawtooth nanostructure between excitation with left- and right-handed circular-polarized fundamental wave. The CD-SHG response of the structure was enhanced significantly by multipolar interactions associated with asymmetric local field distribution and surface charge distribution. In additional to nonzero chiral optical susceptibility tensor components, the chirality of the structure also leads to different multipolar contributions to the second-order nonlinear process upon LCP or RCP illumination. Our results not only show that SHG allows an accurate determination of the chiral symmetry breaking in materials, but also suggest a way for the design of plasmonic structures with giant chiroptical response via manipulating the higher multipolar interactions through chiral symmetry.

## Methods

### Sample Preparation

The Au-embedded sawtooth gratings were fabricated using an e-beam direct write technique as reported in our previous work^18^. A layer of 250 nm thick polymethyl methacrylate (PMMA) was first spin-coated on the indium-in-oxide (ITO) glass. Then PMMA sawtooth grating templates were obtained by e-beam direct-write using a JEOL 6300 scanning electron microscope (SEM). After, a 50 nm Au was deposited under high vacuum onto the PMMA grating templates using e-beam evaporation at normal incidence. Finally, Au sawtooth gratings consisting of И and N unit patterns (viewed from the top of the air/grating/substrate structure) were obtain after a lift-off process.

### Experimental Characterization

For the SHG-CD measurements, a Ti:sapphire laser at a wavelength of 820 nm, pulse width of 200 fs, repetition rate of 76 MHz, and average power of 60 ~ 80 mW was used as the pump radiation. The fundamental beam was focused on the sample using an objective lens to a spot approximately 5 μm in diameter. The wave vector of the laser beam was along the surface normal and its polarization was controlled using a linear Gran-Laser polarizer and a continuously rotating Newport achromatic zero-order quarter waveplate (10RP54-2). The QWP angles of 0, 90, 180 degree, etc correspond to linear input polarization and angles of 45 and 135 degree correspond to RCP and LCP circular input polarizations, respectively. After passing through a collecting objective lens and a band-pass filter (SCHOTT BG39), SHG signal generated from the sample was coupled to a Jobin Yvon SPEX500M spectrometer with the liquid nitrogen (N_2_) cooled Spectrum One CCD head by an optical fiber. The SHG signal was depolarized by the optical fiber before entering the spectrometer to avoid polarization-dependent responses of the grating in our experiment. With the surface normal of the sample coincide with the wave vector of the input beam and the detecting direction of the emitted SHG signals, the experimental setup exhibits no extrinsic optical activity except the handedness of the light polarization vector^1^,^37^. The circular-difference responses were mainly related to the chirality of the sample instead of the sample’s in-plane anisotropy^7^,^38^. For the SHG-CD measurements in reflection geometry, a dichroic mirror (Semrock FF509-Di01 dichroic beam splitter) was used to direct the fundamental laser (820 nm) onto the sample through objective lens, where the reflected SHG signal (410 nm) collected by the same objective lens passing through. Detailed information on the chiral properties of the sawtooth gratings was then obtained by comparing the SHG line shapes measured in either transmission or reflection geometries. Similar to that in our previous work^18^, we define the forward and backward excitation direction as laser pulses enters the sample with incidence on the glass substrate and the Au grating side, respectively.

### Numerical Simulations

Numerical simulations were performed with a commercial software, Lumerical FDTD Solutions, and a free open-source software package applying discrete-dipole approximation (DDA)^39^, DDSCAT 7.3. The Au sawtooth gratings were modeled as a 2D periodic target with a unit cell of 450 × 900 nm^2^. The local-field distributions at 820 nm on the sawtooth gratings were calculated by DDA simulation with an inter-dipolar separation of 5 nm. Only the 50 nm Au grating and a 65 nm ITO layer were considered, while the glass substrate was not included in the DDA simulation. The transmittance of the structure in vacuum was calculated with FDTD simulation for LCP or RCP light from 650 nm to 900 nm. The mesh step was 7.9 nm and the samples were modeled as a three-layer system containing the 50 nm Au grating, an 65 nm-thick ITO layer, and an 1 μm-thick silica substrate in the FDTD simulation. The dielectric constants for gold were taken from experimental data in both simulations^40^.

## Additional Information

**How to cite this article**: Su, H. *et al.* Multipolar Effects in the Optical Active Second Harmonic Generation from Sawtooth Chiral Metamaterials. *Sci. Rep.* **6**, 22061; doi: 10.1038/srep22061 (2016).

## Supplementary Material

Supplementary Information

## Figures and Tables

**Table 1:** Fitting values of the isotropic combinations of the NRT components in the mirror-N sawtooth grating.

Tensor Component	Value	Magnitude	Chirality
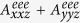	1	1	Achiral
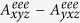	−0.07	0.07	Chiral
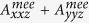	−0.24 + 0.04i	0.24	Chiral
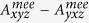	−0.20–0.16i	0.25	Achiral
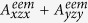	−0.36–0.39i	0.53	Chiral
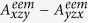	1.00–0.07i	1.00	Achiral
