# Supplementary material for: Multipolar Effects in the Optical Active Second Harmonic Generation from Sawtooth Chiral Metamaterials
Source: Sci Rep. 2016 Feb 25;6:22061. doi: 10.1038/srep22061 (PMC4766511; doi:10.1038/srep22061)
Supplement: Supplementary Information [file srep22061-s1.pdf]

Multipolar Effects in the Optical Active Second Harmonic Generation from Sawtooth Chiral Metamaterials

Huimin Su[1,3], Yuxiang Guo[1], Wensheng Gao[1], Jie Ma[2], Yongchun Zhong[2], Wing Yim Tam[1], C. T. Chan[1], Kam Sing Wong[1*]

[1] Department of Physics, The Hong Kong University of Science and Technology, Hong Kong, P. R. China

[2] Key Laboratory of Optoelectronic Information and Sensing Technologies of Guangdong Higher Education Institutes, Jinan University, Guangzhou 510632, P. R. China

[3] Department of Physics, The South University of Science and Technology of China, Shenzhen 518055, P. R. China

*Correspondence to: phkswong@ust.hk

Supporting Information.

Schematic of the sample and experimental configuration

The Au sawtooth gratings were fabricated using an e-beam direct write technique as reported in our previous work[1].

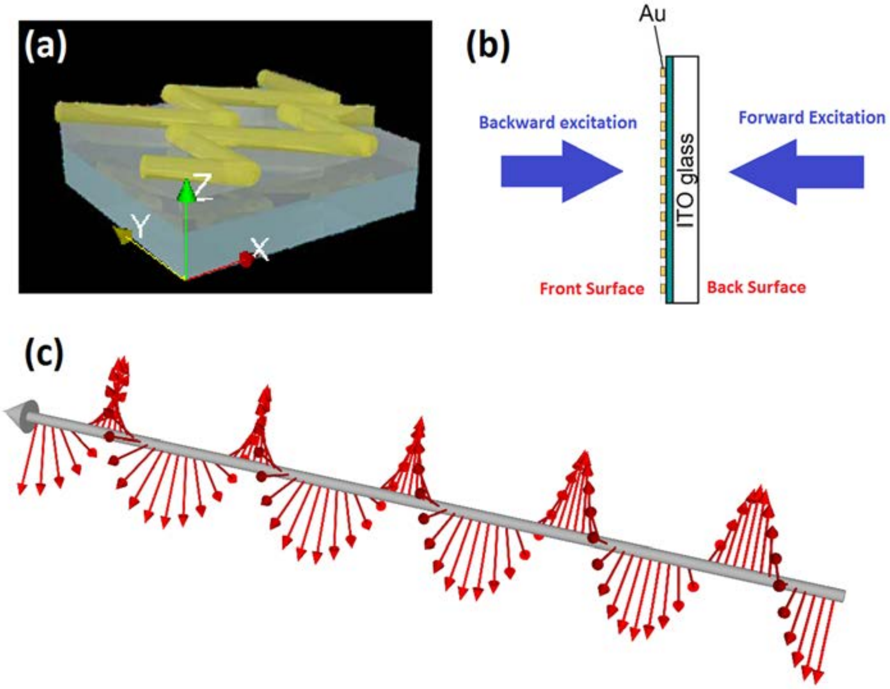

Figure S1. (a) Schematic of the plasmonic sawtooth structure. (b) The definition of backward excitation and forward excitation, front surface and back surface. (c) Definition of RCP light in this work: the grey arrow shows the propagation direction, and the red vectors indicate the electric field, which rotate clockwise as time increase from the point of view of the receiver. Please note that this convention of handedness is different from that used in our previous work on the linear CD measurement (Ref. 18).

Linear optical properties of the N-type sample

The CDs of N and mirror-N gratings were measured with a home-made optical platform[2]. Circular polarized light from 400 nm to 900 nm was generated with a polarizer and a superachromatic, zero-ordered quarter waveplate (Astropribor APSAW-5). The transmitted light was measured by an Ocean Optics USB4000 spectrometer. Similar to that in our previous work[1], we define the forward and backward excitation direction as laser pulses enters the sample with incidence on the glass substrate and the Au grating side, respectively (Fig. S1b-c).

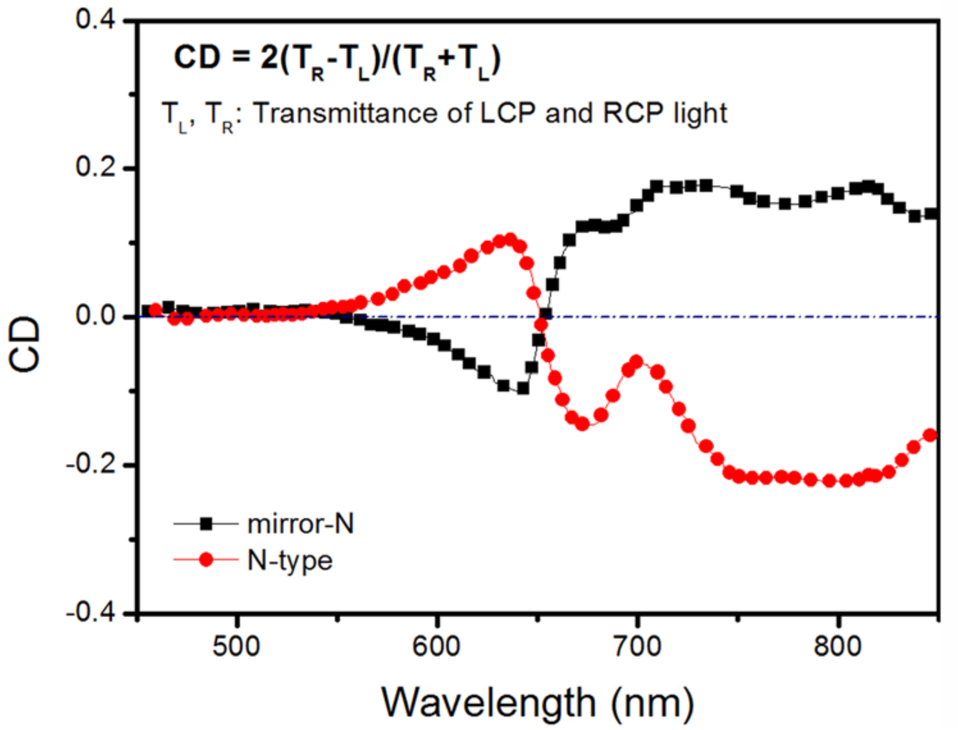

Figure S2. The measured CD of the mirror-N and N-type gratings with backward excitation.

The average ohmic power loss density was calculated by $1/2\,\langle \boldsymbol{J}^{*} \cdot \boldsymbol{E} \rangle$ at the wavelength of 820nm upon LCP and RCP illumination, where $\boldsymbol{J}$ is the current density and $\boldsymbol{E}$ is the electric field in the metal.

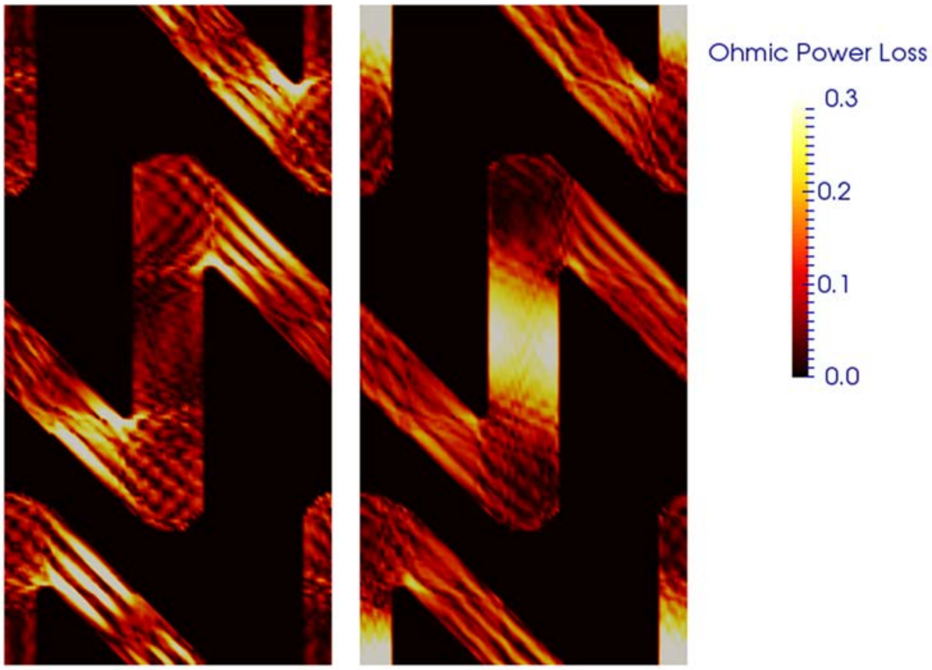

Figure S3. Calculation on the ohmic power loss in the N-type grating with backward excitation upon LCP (left) and RCP (right) illumination at 820 nm.

<u>Power dependence measurement of SHG signal</u>

The dependence of SHG intensity on the average power of the circularly polarized pump laser is shown in Fig. S4. A quadratic dependency of $I_{2\omega} \propto {I_{\omega}}^{K}$ is obtained from the least square fitting with the exponential factor $K = 2.02 \pm 0.04$.

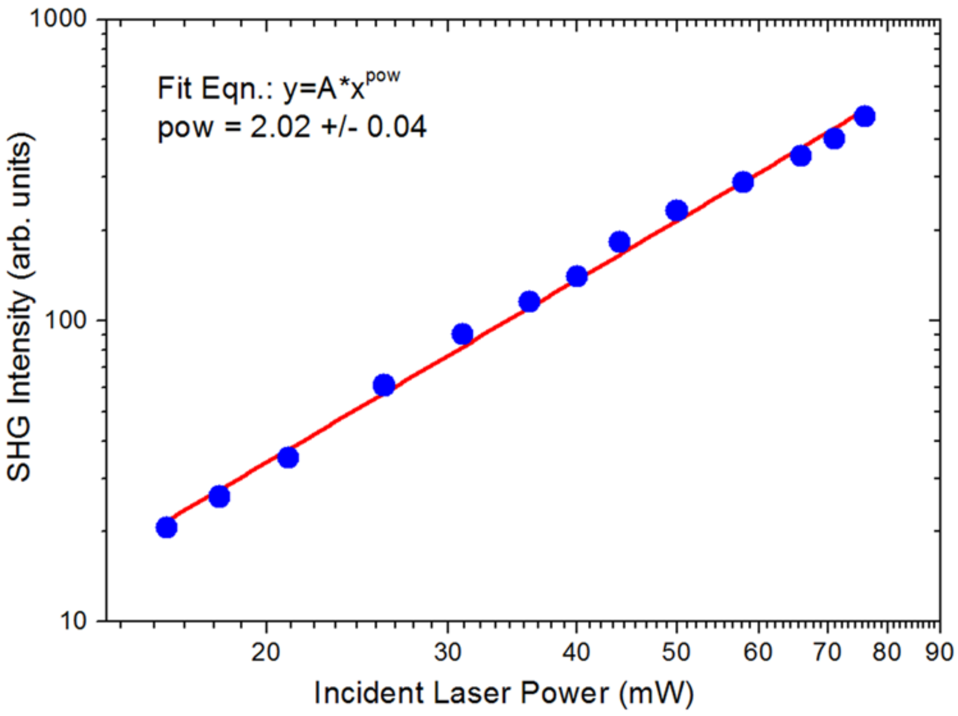

Figure S4. The quadratic dependence of the measured SH intensity on the incident laser power for the N-type grating upon forward LCP (820nm) excitation.

Nonlinear response tensor model (NRT)

The nonlinear polarization and magnetization up to the first-order magnetic-dipole transitions are:

$$P_i(2\omega) = A_{ijk}^{eee} E_j E_k + A_{ijk}^{eem} E_j B_k, \quad \text{(S1)}$$

$$M_i(2\omega) = A_{ijk}^{mee} E_j E_k, \quad \text{(S2)}$$

where A^{eee} refers to the electric-dipole only interactions, A^{eem} refers to magnetic interactions at the fundamental frequency, and A^{mee} refers to magnetic interaction at the SH frequency. Both

the magnetic dipole and electric quadrupole effects are included in the effective magnetic tensors, due to the difficulty in separation between their contributions to the SH signals. The nonvanishing tensor components for C_2 symmetry group are zzz, zxx, zyy, $zxy = zyx$, $xxz = xzx$, $xyz = xzy$, $yxz = yzx$, $yyz = yzy$, where z is the surface normal. Only the nonvanishing tensor components were involved in the calculation. (Note that the tensor A_{ijk}^{eem} does not obey intrinsic permutation symmetry in the indices *j* and *k* listed above[3], i.e. $A_{ijk}^{eem} \neq A_{ikj}^{eem}$.) In addition, the magnetic field $\boldsymbol{B}(\omega)$ depends linearly on the electric field $\boldsymbol{E}(\omega)$. Now we analyze these 3 tensors for a quasi-2D sample in C_2-symmetry. With the two-fold rotation axis along the surface normal (*z*-axis), every tensor component where the sum of the number of *x*'s and y's in its index is odd must be zero. And the nonvanishing tensor components of tensor A^{eem} and A^{mee} are same as those of the tensor A^{eee}. The sawtooth grating layer also have a mirror plane in the *x*-*y* plane, which have no further influence on the A^{eem} and A^{mee} tensor. But for the A^{eee} tensor, all the remaining components will be cancelled under reflection. However the existence of the glass substrate enables the observation of SHG arising from pure electric dipole interaction. According to the radiative properties of electric dipoles and higher multipoles, all the A^{mee} components change in signs when the measurement direction is reverted. And the samples with 'N' and 'И' patterns are related by the coordinate transformation $x \rightarrow -x, y \rightarrow y, z \rightarrow z$. Thus every component of tensor A^{eee} (A^{eem} and A^{mee}) with odd (even) numbers of *x*'s in its index change in sign with regard to those in the mirror structure. Finally, we ignore the terms in Eq. (S1) with $B_z(\omega)$ component, since neither *x*- and *y*-polarized EM field can drive an effective magnetic field along the z direction in our experiments. Table S1 list the expansion coefficients of Eq. (2) in various configurations based on the NRT components of the mirror-N samples. The

change of signs of the tensor components to different signals was associated with the radiative properties of various multipoles and the rotational symmetry of the samples.

Table S1. The expansion coefficients of the detected SH signal as function of NRT components in each configuration. N-: N-type sample, M-: mirror-N sample, -T: transmission collection, R-: reflection collection, -x: x-polarized SH signal ($i = x$), -y: y-polarized SH signal ($i = y$). The degeneracies of $A_{ijk} = A_{ikj}$ for A^{eee} and A^{mee} have been omitted.

Geometry	A_{ixz}	A_{iyz}
$\mathrm{M-T-x}$	$A^{eee}_{xxz} + A^{eem}_{xzy} + A^{mee}_{yxz}$	$A^{eee}_{xyz} - A^{eem}_{xzx} + A^{mee}_{yyz}$
$\mathrm{M-T-y}$	$A^{eee}_{yxz} + A^{eem}_{yzy} - A^{mee}_{xxz}$	$A^{eee}_{yyz} - A^{eem}_{yzx} - A^{mee}_{xyz}$
$\mathrm{M-R-x}$	$A^{eee}_{xxz} + A^{eem}_{xzy} - A^{mee}_{yxz}$	$A^{eee}_{xyz} - A^{eem}_{xzx} - A^{mee}_{yyz}$
$\mathrm{M-R-y}$	$A^{eee}_{yxz} + A^{eem}_{yzy} + A^{mee}_{xxz}$	$A^{eee}_{yyz} - A^{eem}_{yzx} + A^{mee}_{xyz}$
$\mathrm{N-T-x}$	$A^{eee}_{xxz} + A^{eem}_{xzy} + A^{mee}_{yxz}$	$-A^{eee}_{xyz} + A^{eem}_{xzx} - A^{mee}_{yyz}$
$\mathrm{N-T-y}$	$-A^{eee}_{yxz} - A^{eem}_{yzy} + A^{mee}_{xxz}$	$A^{eee}_{yyz} - A^{eem}_{yzx} - A^{mee}_{xyz}$
$\mathrm{N-R-x}$	$A^{eee}_{xxz} + A^{eem}_{xzy} - A^{mee}_{yxz}$	$-A^{eee}_{xyz} + A^{eem}_{xzx} + A^{mee}_{yyz}$
$\mathrm{N-R-y}$	$-A^{eee}_{yxz} - A^{eem}_{yzy} - A^{mee}_{xxz}$	$A^{eee}_{yyz} - A^{eem}_{yzx} + A^{mee}_{xyz}$

The experimental results were then normalized to unity and fitted to the NRT model of Eq. (2). For the C_2 symmetry, the values of tensor components depend on the choice of x- and y-direction of the coordinate system. Accordingly, we examine the isotropic combinations of tensor components instead: xxz + yyz, xzx + yzy, and xyz − yxz in Table 1.

1. Gao, W.; Ng, C. Y.; Leung, H. M.; Li, Y.; Chen, H.; Tam, W. Y., Circular dichroism in single-layered gold sawtooth gratings. *J. Opt. Soc. Am. B* **2012,** *29* (11), 3021-3026.
2. Gao, W.; Leung, H. M.; Li, Y.; Chen, H.; Tam, W. Y., Circular dichroism in double-layer metallic crossed-gratings. *Journal of Optics* **2011,** *13* (11), 115101.
3. Kauranen, M.; Verbiest, T.; Persoons, A., Second-order nonlinear optical signatures of surface chirality. *Journal of Modern Optics* **1998,** *45* (2), 403-423.
